# Effects of arginine vasopressin on musical working memory

**DOI:** 10.3389/fpsyg.2013.00712

**Published:** 2013-10-17

**Authors:** Roni Y. Granot, Florina Uzefovsky, Helena Bogopolsky, Richard P. Ebstein

**Affiliations:** ^1^Department of Musicology, The Hebrew University of JerusalemJerusalem, Israel; ^2^School of Social Work and Social Welfare, The Hebrew University of JerusalemJerusalem, Israel; ^3^Psychology Department, National University of SingaporeSingapore, Singapore

**Keywords:** arginine vasopressin, AVPR1A, musical memory, arousal, digit-span, verbal memory, working memory

## Abstract

Previous genetic studies showed an association between variations in the gene coding for the 1a receptor of the neuro-hormone arginine vasopressin (AVP) and musical working memory (WM). The current study set out to test the influence of intranasal administration (INA) of AVP on musical as compared to verbal WM using a double blind crossover (AVP—placebo) design. Two groups of 25 males were exposed to 20 IU of AVP in one session, and 20 IU of saline water (placebo) in a second session, 1 week apart. In each session subjects completed the tonal subtest from Gordon's “Musical Aptitude Profile,” the interval subtest from the “Montreal Battery for Evaluation of Amusias (MBEA),” and the forward and backward digit span tests. Scores in the digit span tests were not influenced by AVP. In contrast, in the music tests there was an AVP effect. In the MBEA test, scores for the group receiving placebo in the first session (PV) were higher than for the group receiving vasopressin in the first session (VP) (*p* < 0.05) with no main Session effect nor Group × Session interaction. In the Gordon test there was a main Session effect (*p* < 0.05) with scores higher in the second as compared to the first session, a marginal main Group effect (*p* = 0.093) and a marginal Group × Session interaction (*p* = 0.88). In addition we found that the group that received AVP in the first session scored higher on scales indicative of happiness, and alertness on the positive and negative affect scale, (PANAS). Only in this group and only in the music test these scores were significantly correlated with memory scores. Together the results reflect a complex interaction between AVP, musical memory, arousal, and contextual effects such as session, and base levels of memory. The results are interpreted in light of music's universal use as a means to modulate arousal on the one hand, and AVP's influence on mood, arousal, and social interactions on the other.

## Introduction

Music is a rich and varied sociocultural phenomenon whose roots may be biological: as a form of courtship and signal of fitness (Darwin, 1871; Miller, 2000); as an essential part of mother-infant communication paving the way to emotional regulation, social behavior and language (Dissanayake, 2000; Malloch and Trevarthen, 2009), or as a unique way of achieving synchronization and group cohesion (Huron, 2001; Kirschner and Tomasello, 2009). It is therefore intriguing that previous studies (see below) have found an association between genetic make-up, musical memory, itself found to be heritable with *h*^2^ estimated at 0.42–0.80 (Drayna et al., 2001; Pulli et al., 2008), and neuro-hormones heavily involved in social behaviors and emotional states. While it is clear that music can have deep emotional effects and that its processing relies heavily on memory, why or how should such neuro-hormones modulate musical memory, and whether this neuro-mollecular underpinning is unique to music or shared by other auditory-vocal systems such as language—remains absolutely unknown. In the current study we attempt to make the first step toward answering some of these questions.

### Musical working memory, the neuro-hormone arginine vasopressin, and genetics

In a previous study we found an intriguing association between musical working memory (WM) (Granot et al., 2007) and variations of the gene coding for the 1a receptor of the neuro-hormone arginine vasopressin (AVP). In that study we reported on an association between promoter repeats in the arginine vasopressin 1a receptor (AVPR1a) and serotonin transporter *SLC6A4* (HTTLPR) genes, and scores on a number of musical and phonological WM tests. The strongest associations were found when both AVPR1a RS1 and RS3 microsatellite regions were grouped by the HTTLPR short or long promoter repeat in relation to two well-established tests for tonal memory: Gordon's “Musical Aptitude Profile” (MAP, 1965) and the interval subtest (“same contour”) from the “Montreal Battery for Evaluation of Amusias,” (MBEA, Peretz et al., 2003), as well as in relation to a phonological test devised for that study. In addition, we found a *negative* correlation between total repeat length of RS1 and RS3 base pairs and the scores in the MBEA test. These same two genes were found by our group (Bachner-Melman et al., 2005) to be associated with dancing, which can be considered as a closely related phenotype.

A replication of our previous study was achieved by Ukkola et al. (2009) who genotyped 298 individuals from 19 Finnish families. In their study musical aptitude was tested via the Karma test (Karma, 2007) which relies on memory for pitch patterns, and Seashore's pitch and time discrimination subtests from his battery of musical abilities (Seashore et al., 1960). As in Granot et al. (2007), a strong association between memory for music and AVPR1a RS1 and RS3 promoter repeats was observed. In addition, those scoring high on the music tests were also more prone to score high on music creativity, in itself also found to be, in that study, highly heritable (*h*^2^ = 0.84). When tested for a direct association between SLC6A4 and the musical test, a weak association was found only with the Karma test, with no reported results of Gene × Gene tests similar to those described in Granot et al. (2007).

### AVP and social and emotional behavior

AVP has been strongly implicated in social behavior, particularly in males. Social behaviors and emotional states typically associated with AVP include pair-bond formation, courtship, aggression, fear and anxiety, and increased vigilance and arousal. Most of these effects have been shown in rodents (for a review see, Young and Wang, 2004; Insel, 2010), but recent studies have found some similar effects in humans (Donaldson and Young, 2008; Heinrichs et al., 2009; Bos et al., 2012). Our group (for a review see Ebstein et al., 2010) has shown an association between AVPR1a receptor gene and a number of social behaviors in various clinical and non-clinical populations including measures of social stress (Ebstein et al., 2009), eating behavior (Bachner-Melman et al., 2004), altruism (Knafo et al., 2008) dance (Bachner-Melman et al., 2005), and autism (Yirmiya et al., 2006).

Complementing these genetic studies are a growing number of pharmacological studies showing that neuropeptides, such as vasopressin, which gain access to the human brain after intranasal administration (INA) (Born et al., 2002), modify human social behaviors. Thompson et al. (2004) found that INA of AVP stimulates, in males, agonistic facial motor responses to neutral same-sex faces, whereas it enhances affiliative responses in women. Similarly, under AVP administration, male, but not female responded with agonistic facial responses to pictures of unfamiliar men displaying happy expressions and also rated them as less friendly. These were accompanied by heightened autonomic responses and anxiety levels (Thompson et al., 2006), and may reflect sex specific stress coping strategies. Consistent with this account, Uzefovsky et al. (2012) reported reduced ability of males receiving AVP to correctly identify negative, (but not positive) emotions in “Reading the Mind in the Eyes” test (Baron-Cohen et al., 2001), in response to photos of males as compared to females.

Some interaction between emotional information, memory and AVP has also been reported. Emotional faces elicited, in the presence of AVP, better encoding as compared to neutral ones. However, this was limited to familiarity ratings with no comparable effects for recognition (Guastella et al., 2010). Finally, Shalev et al. (2011) showed that AVP increased levels of cortisol and heart rate only in conditions of social stress as compared to physical stress or mental load.

It has been proposed that these social/emotional effects of AVP are mediated in rodents by vasopressin V1a receptors in the lateral septum, hypothalamus, bed nucleus of the stria terminalis, hippocampus, amygdala, and brainstem (Loup et al., 1991; Young et al., 1999). In humans, Zink et al. (2010) used fMRI to examine the influence of AVP on processing human faces with different emotional expressions, focusing on the medial Prefrontal Cortex (mPFC) known to regulate amygdala activity. The results indicate that the fear/aggression social behaviors related to AVP, are mediated by its influence on the feedback loop between the amygdala and mPFC.

### AVP and memory

A large number of studies show memory enhancing effects of AVP administration on hippocampus dependent memory in rodents, as seen in active or passive avoidance tasks (Feany, 1996), in social recognition (Bielsky and Young, 2004), and in spatial memory (Alescio-Lautier and Soumireu-Mourat, 1999). Conversely, mice with null mutation in the vasopressin V1a receptor display social recognition impairments (Engelmann and Landgraf, 1994; Landgraf et al., 1995).

In humans, the memory effects of AVP are more controversial. Whereas a number of studies have shown enhanced memory for verbal memory (e.g., words, numbers, sentences, stories) and for visual information (e.g., visual patterns, maze tests), others have found no such effects (for a review see: Caldwell et al., 2008). For a summarizing table of AVP effects on memory and attention in humans as reflected in studies published in 1981–1998, see Table 1 in Born et al. (1998). This table highlights the large variability in the examined experimental variables including number of subjects, gender, dosage, administration protocol, and the specific administered memory or cognitive tests.

On the basis of the neuroanatomy of the AVP system, Born et al. (1998) suggested that AVP mediates its memory effects via the hippocampus and hence postulated that some of the variability in the results seen in their Table 1 can be explained by differences in the presumed underlying memory system supporting performance in the various tests.

Other researchers, however, have suggested that the beneficial memory effects of AVP are mediated by non-specific cognitive arousal (Snel et al., 1987; Gais et al., 2002), or by specific enhanced sensitivity to deviance, novelty and violation of expectations which mark specific salient items for memory. This interpretation is compatible with the consistent enhancement, in the presence of AVP, of Event-related brain potential (ERP) components indicative of such processes such as the N1, MMN, and P3 (Born et al., 1998).

### Musical and verbal working memory

The memory literature defines WM as comprising of both a “storage” of temporarily accessible limited amount of information short term memory (STM) as well as the cognitive processes that maintain and/or manipulate the held information (Cowan, 2005). Components of these cognitive processes include maintenance operations, rehearsal, shifts of attention across items within the held information and retrieval mechanisms. However, their exact nature, and the degree to which they are basic or reflect more complex strategies beyond STM, is a matter of debate (Jonides et al., 2008).

One influential model of WM is Baddeley's model of verbal WM (e.g., Baddeley, 1986, 1992). According to this model verbal WM is presumed to include three components: a phonological store that holds the verbal information for a few seconds, a phonological rehearsal component based on subvocalization of the to-be remembered items, and an executive component. Clearly, all three components are relevant to holding musical information in short term memory, including the notion of subvocal rehearsal as shown by Koelsch et al. (2009). Applying this model to music, begs the question of the degree of overlap or distinction between memory for pitch and memory for speech—mainly whether there is a different storage component for tonal as compared to verbal information. At least partial dissociation has been shown by Deutsch (1970). In that study Deutsch showed that retention of a single pitch to be compared with a second pitch following a delay of a few seconds is heavily influenced by the type of information presented during this delay. Whereas intervening pitch information clearly degrades memory, speech information does not (see also Salamé and Baddeley, 1989). As a result a separate “tonal-loop” analogous to Baddeley's proposed phonological loop has been proposed (Pechmann and Mohr, 1992; Berz, 1995).

Other studies, however, point to at least some components of auditory WM which are common to holding and manipulating both pitch and verbal information. These studies have either shown some interference of pitch information on verbal memory (Iwanaga and Ito, 2002; Alley and Greene, 2008) or more generally positive influence of musical training on verbal WM (Ho et al., 2003; Fujioka et al., 2006; Bugos et al., 2007; Roden et al., 2012).

A significant support for the idea of shared rather than independent components of tonal and verbal WM is obtained via brain studies showing largely overlapping neural networks supporting WM for both types of auditory information. In general, tonal memory is supported by a wide network including primary and secondary auditory areas more right lateralized, the supramarginal gyrus particularly on the left, and dorsolateral inferior frontal areas, more clearly seen under heavy memory load conditions (Zatorre et al., 1994; Griffith et al., 1999; Gaab et al., 2003). Premotor areas, the cerebellum, basal ganglia and the thalamus are activated when subvocal tonal rehearsal is explicitly required (Hickok et al., 2003; Koelsch et al., 2009). Importantly a direct comparison of memory for verbal syllables and sung pitches under rehearsal and under suppression elicited largely overlapping brain areas consistent with this network (Schulze et al., 2011).

Although Baddeley's model has been especially influential in the music cognition literature, a more recent approach may also be relevant to the current study. Briefly, this approach assumes a domain-general model of WM, where, in contrast with Baddeley's model there is no distinction between long term memory (LTM) and STM (e.g., Cowan, 2001, 2005; Oberauer, 2002). Rather, memory representations of items are activated, either by incoming sensory input or by volition, thus becoming available for attentional selection. Importantly, capacity limits estimated to range from 1 to 4 (depending on the exact model) individual items, or chunks, are assumed to depend on the attentional selection mechanisms. In this model the main “bottleneck” is the ability to shift focus of attention among a number of activated representations as found in serially complex sequences such as music. Hence factors influencing attention such as general arousal levels (Sarter et al., 2001) may have significant effects on memory.

### The experimental rationale

This introduction maps a complex set of components related to musical WM on the genetic, molecular, and cognitive level. Musical WM shares a number of components with verbal WM, it is heritable, and scores of WM are associated with AVPR1a haplotypes. AVP has been shown to influence verbal WM, but not always, and there is no agreement whether its influence is direct and specific (e.g., through brain regions heavily involved in memory such as the hippocampus), or mediated by general factors such as anxiety, attention and arousal. Hence we set out to study the direct effects of AVP on musical WM using INA of AVP posing these three questions (1) whether similar or different effects of AVP would be seen on verbal and musical WM, (2) whether these effects would be toward enhancing or degrading WM, and (3) whether they could more easily be explained through direct and specific influence of AVP on WM or rather through second order, non-specific effects related to mood, arousal and attention.

Since AVP has been to shown to influence not only memory directly, but also other second-order factors which could potentially influence memory such as anxiety, social stress, general arousal, (Ashby et al., 2002; Mitchell and Phillips, 2007), we asked our subjects to fill out questionnaires related to these factors. Both anxiety state and trait have been shown to influence WM albeit in complex ways, depending on the type of memory (e.g., verbal vs. visual), the type of anxiety (e.g., social or other), and the degree of distracting information. In general degrading effects have been shown, mostly explained by the depleting effects of worry on WM capacity (more consistent with Baddeley's, 1992 WM model) or reduction of attentional focus on current task as suggested more recently Eysenck et al. (2007) (more consistent with Cowan's, 2001 WM model).

We applied a double blind crossover (AVP—placebo) design in which 25 healthy males with little or no musical training were randomly assigned to one of two groups: The first group was exposed to intranasal dosage of 20 IU of AVP in the first session and a similar dose of 20 IU of Placebo (saline water) in a second session 1 week apart. The second group received the reverse order of drugs: Placebo in the first session and AVP in the second. In each session subjects completed a set of questionnaires related to their attitude and reactivity to music (the “Brief Musical Experience Questionnaire,” Werner et al., 2006), to anxiety (the Beck Anxiety Inventory (BAI); Beck and Steer, 1990); and to mood (a visual analog adjusted scale based on items from the “Positive and Negative Affect Scale” (PANAS), Tellegen et al., 1988). They then performed four memory tests. These included two musical memory tests: the tonal subtest from Gordon's MAP (1965); the interval subtest (same contour, in-key deviations) from the MBEA (Peretz et al., 2003); and two tasks of verbal WM (the digit span test both forward and backward from the Wechsler Adult Intelligence Scale III, Wechsler, 1997).

## Materials and methods

### Participants

A total of 50 healthy male students (mean age ± *SD*, 25.08 ± 2.89) from the Hebrew University of Jerusalem participated in the study. All subjects had little or no musical training (see Table 1). Only male participants were recruited due to the many findings that point to a male-specific effect of AVP (for a review see: Heinrichs and Domes, 2008; Insel, 2010). Only participants with no history of psychiatric or endocrine illness (by self-report), non-smokers, and not using medication on a regular basis were included in the study. Participants were asked to abstain from food, drink (other than water) and physical exercise during 90 min preceding the experiment. Twenty five participants were randomly assigned to receive AVP in the first session and Placebo in the second session (VP = Vasopressin—Placebo Group) and the remaining 25 received Placebo in the first session and AVP in the second (PV = Placebo—Vasopressin Group). Participants were informed at the time of recruitment that the experiment evaluates the effects of a hormone on memory for music. Participants were paid a total sum of 200 NIS (~50 U.S.$) for their participation in both sessions. The study was approved by the S. Herzog Hospital IRB committee and the Israeli Ministry of Health.

**Table 1:** **Age and musical background and attitudes of the subjects**.

	**Group 1 PV** (***n*** = **25**)	**Group 2 VP** (***n*** = **25**)
Age (*SD*)	23.6 (5.4)	25.6 (3.3)
Years of musical training (*SD*)	1.86 (2.2)	1.24 (2.4)
**SELECTED ITEMS FROM BMEQ**		
(2) I frequently hear songs in my head	4.24 (0.92)	4.12 (0.99)
(10) It's hard for me to keep the beat when dancing	2.28 (1.1)	2.48 (1.26)
(14) There's nothing more powerful than singing a beloved song with other people	2.88 (1.09)	2.88 (1.18)
(30) I have a good sense of pitch	3.12 (1.24)	2.32 (1.29)
(31) I really get “lost” in the depth of my concentration on music	2.6 (0.82)	3.12 (1.18)
(32) I often find myself swaying in tune with the music to which I am listening	3.4 (1.26)	3.76 (1.07)
(42) Music helps me not feel so lonely	3.04 (1.17)	2.88 (1.03)
(51) Music can influence my emotions	4.12 (1.07)	4.12 (0.78)

T-tests on age (t = −1.6, df = 48, p = 0.114); years of training (t = 0.95, df = 48, p = 0.35); BMEQ—Brief Musical Experience Questionnaire (first session): for all items p > 0.24 adjusted (Bonferroni correction) for multiple testing.

### Design and procedure

Testing was carried out at the Music Cognition Lab at the Hebrew University. Participants were scheduled for testing within a fixed time-window (between 16:00 and 19:00 h) to control for effects of circadian changes in endogenous AVP secretion. The session lasted for 75 min and was repeated 1 week later.

Prior to administering AVP or saline control, participants were informed that they would receive in one session the hormone and in another the placebo, but that neither they nor the experimenter could know which is which. They were thoroughly informed about the nature of the hormone and its possible side effects, and were then asked to read and sign the consent form. They were also told they could withdraw from the experiment at any time. None of the participants, however, asked to do so. Twenty IU's of AVP (diluted in 0.9% NaCl, Sigma), or placebo (sterile saline, 0.9% NaCl) were self-administered to both nostrils in the presence of the experimenter by use of a medicine dropper.

During the waiting period of 15 min until the hormone becomes effective, participants filled out a battery of demographic questionnaires, reporting on personal demographics, musical background, habits, musical training, and the Brief Musical Experience Questionnaire (BMEQ) (Werner et al., 2006) in its Hebrew translation. The BMEQ includes 53 questions related to the place of music in the individual's life and her reactivity to it. Subjects were asked to rate on a 1–5 scale how much each statement was true of her experience or behavior (1 = least, 5 = most). Items related to the subject's commitment to music, affective and motional reactions to it, creative tendencies and the degree to which music is associated in her life with social uplift.

Following 15 min from intranasal administration (AVP or placebo) subjects were asked to fill out two questionnaires which provide self-reported information on factors known to influence WM on the one hand, and known to be influenced by AVP on the other. These included information on mood, arousal, attention, and anxiety as obtained by (1) the BAI (Beck and Steer, 1990) and (2) a visual analog scale based on items from the adjusted PANAS, (Tellegen et al., 1988). (1) The BAI inventory includes 21 multiple choice questions relating to physiological and emotional states indicative of levels of anxiety. Subjects were asked to rate the degree (0–3) to which each statement reflected their state *within the last week including the day of the experiment*. (2) The adjusted PANAS included 20 statements describing different emotional states related to positive and negative affects (e.g., “At this moment I feel happy”), arousal (e.g., “At this moment I feel alert”), attention (e.g., “At this moment I feel attentive”), and worry (e.g., “At this moment I feel scared”). Subjects rated how much each statement described their mood at the time, by marking a position along a continuous line between two end points ranging from “completely agree” to “completely disagree.”

Twenty to twenty-five min after administration of the substance, participants performed four memory tests in a randomized order: (1) the tonal imagery subtest from Gordon's MAP (1965), (2) the interval subtest from the MBEA (Peretz et al., 2003) and the digit span test—both, (3) forward (FDS), and (4) backward (BDS) from the Wechsler III intelligence test in its' Hebrew version (Wechsler, 1997). The same order of tests was maintained in the second session. In addition, since these tests are standardized tests, the items in each of the tests were presented in the two sessions in the same exact order despite some likelihood of learning effects.

(1) In the MBEA battery, tonal melodies are presented in pairs with 50% of the melodies repeating exactly (“same” melodies) and the remaining 15 melodies repeating with a change in a single pitch (“different”). In the interval subtest (the only subtest from the battery used here), “different” melodies retain the contour of the original melody and only one interval changes such that the new pitch remains within the original key (the most difficult of the tonal tests of this battery). Subjects' task was to decide whether the two melodies in each pair are same or different. Although no reliability measures are given for subtests of the battery, the correlation of the entire battery over test–retest is reported to be *r* = 0.75 and it is positively correlated with the entire Gordon battery at *r* = 0.53 (Peretz et al., 2003).
(2) The tonal imagery subtest of Gordon's MAP consists of 40 pairs of a musical tonal or modal phrases followed by a “musical answer,” both recorded by a real rather than synthesized violin playing. The musical answer is either a variant of the target melody in which case it is considered “similar,” or the musical answer is a different melody. The instructions ask the subject to imagine that the extra notes in the answer “are not really being played” and then to decide whether the musical answer is similar to the target melody or different. Note, that this is quite a different task from the “same-different” comparison task required in the MBEA test, and relies more heavily on WM. Reliability of the Tonal imagery subtest of the Gordon battery is 0.83 (using the split-half procedure) and its validity (correlation with teacher's estimates of musical talent) is 0.81 (Gordon, 1965).
(3, 4) Digit span tests were used here as an indicator of verbal WM. However, unlike the musical tasks, it is a recall task in which participants repeat the digits they have just heard either in the same temporal order (“forward”), or in reverse order (“backward”). The DST was presented by a prerecorded male voice in a moderate pace of ~250 ms per syllable and 0.5 s in between digit words.

All tests were presented via headphones at a comfortable loudness level with each test preceded by written instructions and two training items. The experimental tasks were run within a time window of 50 min presumed to be the time window in which AVP is active in the brain (Born et al., 2002).

## Results

Demographic information on the subjects including information regarding musical training is shown in Table 1. Beyond age and years of musical training we also looked at a selected set of items from the Brief Musical Experience Questionnaire (first session). As seen in Table 1 this selection covers a set of attitudes and behaviors potentially relevant to our study: Self report on musical ability (pitch and rhythm) (items 10 and 30); Reactivity to music (2, 31, 32); Social uplift through music (14) and emotional reactivity to music (42 and 51). *T*-tests on age, years of training and the BMEQ items indicate that our two groups were not statistically different on any of these measures (Table 1).

In order to examine the effects of AVP on memory we ran a separate 2–way repeated measures ANOVA on the raw scores of each of the four tests with Group as a between subjects factor and Session as the within subjects factor. Note that for the VP Group Session 1 = AVP and Session 2 = placebo, whereas for the PV Group the reverse is true: Session 1 = placebo and Session 2 = AVP.

As seen in Tables 2, 3, there are no significant main effects or interactions in the scores of the forward digits span test. In the backward digits span test there is a main Session effect *F*_(1, 48)_ = 4.78 *p* < 0.05 with scores higher in the second as compared to the first session (*M* = 8.58 vs. 9.2, respectively) suggesting some learning has taken place, with no main Group effect, nor a Group × Session interaction. In general these scores are similar to (albeit a bit higher) norms reported for 70 Hebrew University students (see under http://elsc.huji.ac.il/ahissar/links choose Hebrew Reading Norms).

**Table 2:** **Means (and *SD*) of scores on the memory tests**.

	**Digit-span forward**		**Digit-span backward**		**MBEA**		**Gordon**	
	**Session 1**	**Session 2**	**Session 1**	**Session 2**	**Session 1**	**Session 2**	**Session 1**	**Session 2**
Group PV (*N* = 25)^a^	11.16 (1.95)	11.48 (2.10)	8.88 (2.13)	9.36 (2.31)	24.5 (2.63)	25.04 (2.62)	33.37 (4.36)	33.83 (4.72)
Group VP (*N* = 25)	10.92 (2.15)	11.36 (2.65)	8.28 (2.13)	9.04 (2.60)	22.60 (3.27)	22.95 (2.91)	30.04 (5.87)	32.64 (5.27)

a MBEA: N = 20 for the VP Group and N = 24 for the PV Group; Gordon: N = 25 for the VP Group and N = 24 for the PV Group.

**Table 3:** **ANOVA Results (*F*-values) for the four memory tests**.

	**Digit-span forward**	**Digit-span backward**	**MBEA**	**Gordon**
GROUP (PV − VP)	0.94	0.54	6.84^*^	2.93 (*p* = 0.093)
Session (First vs. Second)	2.59	4.78^*^	1.22	6.2^*^
Group × Session	0.65	0.24	0.06	3.04 (*p* = 0.088)

* p < 0.05.

**Figure 1:**
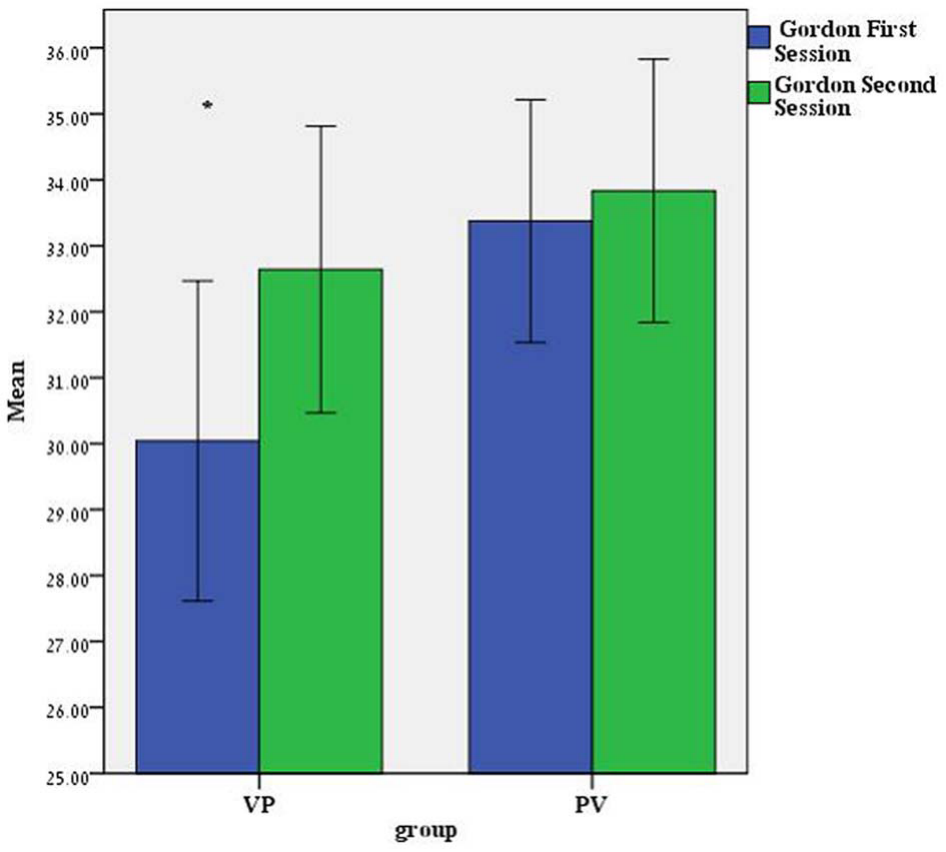
**Mean scores for the Gordon music test in the first and second sessions in the two groups of subjects.** The group receiving Vasopressin in the first session (PV, *N* subjects = 24) performed significantly worse than the group receiving placebo in this session (PV, *N* subjects = 25). No such differences were noted in the second session. ^*^p = 0.029.

Notably, a different pattern of results emerges in the music memory tests. In the MBEA test, scores for the PV Group were higher than for the VP Group [*F*_(1, 48)_ = 6.85 *p* < 0.05 24.95 vs. 22.77, respectively] with no main Session effect nor Group × Session interaction. Results in the Gordon test show a main Session effect *F*_(1, 48)_ = 6.202 *p* < 0.05 with scores higher in the second as compared to the first session (33.22 vs. 31.67, respectively), a marginal main Group effect *F*_(1, 48)_ = 2.93 *p* = 0.093 (*PV* = 33.60 vs. *VP* = 31.34) and a marginal Group × Session interaction *F*_(1, 48)_ = 3.041 *p* = 0.88. As seen in Figure 1, the difference between the groups is significant (One Way ANOVA) in the first session *F*_(1, 48)_ = 5.05 *p* < 0.05 but not in the second session (*p* = 0.41). It would therefore seem that in the music tests there is some interaction between the AVP effects and the order of the sessions such that the AVP effects are stronger in the first as compared to the second session.

We next carried out a second level analysis following the significant findings with the music tests. We first repeated the analysis on the MBEA and Gordon scores adding Years of Musical Training (Training) as a covariate. Pearson correlations between Training and tests scores range from 0.37 (highest) for Gordon scores in the first session to 0.19 (lowest) for the Gordon scores in the second session. For comparison, Training correlates with the digit span scores very poorly (0.11 highest to −0.02 lowest). The ANCOVA results show that while training is a significant factor [*F*_(1, 40)_ = 6.03, *p* < 0.05 and *F*_(1, 45)_ = 5.25 *p* = 0.061 for the MBEA and Gordon scores, respectively], there are no significant interactions of Training with the factors of Group or Session or both. Therefore, training alone cannot explain the pattern of response we see.

In order to probe further the degrading effects of AVP on musical memory seen in the first session, we asked whether these effects are mediated by some interaction between AVP, the musical memory demands and anxiety and mood variables. These variables were probed because they have been associated with AVP on the one hand (see “AVP and social and emotional behavior” in the Introduction) and with memory on the other (Lupien et al., 2007; Joëls and Baram, 2009). Hence we examined whether scores of mood and anxiety were affected by AVP (given that they were collected about 15 min post INA—more or less the time when AVP could become effective), and whether they were in any way correlated with memory scores.

For the mood scales scores (adjusted PANAS) we first performed a PCA in order to reduce the number of scales (20) to a smaller set. Table 4 presents the loadings (varimax rotation, raw) on the first 4 factors (eigen values > 1) which explain together 69% of the variance in the PANAS scores across both sessions. Very similar results are obtained when only scores of the first session are entered into the PCA. Factor 1 has high positive loadings on negative affects: sadness, nervousness, and hostility. Factor 2 in contrast loads mostly on positive and energetic affects such as happiness and excitement. Factor 3 has a high positive loading on calmness and high negative loading on tension hence seems to reflect positive valence with low arousal. Finally factor 4 has high loadings on descriptors related to attentiveness and alertness. To simplify the analyses we selected from each category the scale that had the highest loading in each factor: Aggressive (0.88 on Factor 1); Happy (0.85 on Factor 2); Tense (0.85 on Factor 3); and Focused (0.81 on Factor 4).

**Table 4:** **Factor loadings of the PCA on adjusted PANAS scale**.

	**FACTOR 1**	**FACTOR 2**	**FACTOR 3**	**FACTOR 4**
Happy	−0.21846	**0.846964**	−0.17422	0.076334
Calm	−0.16684	0.169529	−**0.81902**	0.262332
Tense	0.285827	0.093084	**0.852169**	0.052898
Gloomy	**0.780943**	−0.09901	0.269818	−0.13455
Upset	0.583492	0.029475	0.403259	0.054034
Enthusiastic	0.132341	**0.707605**	0.043296	0.214582
Relaxed	−0.05818	0.117599	−0.7656	0.306979
Sad	**0.738563**	−0.01434	0.030247	−0.14739
Distressed	**0.736447**	−0.01134	0.264477	0.247942
Downhearted	**0.846428**	−0.11102	−0.12472	−0.11585
Alert	−0.23053	0.263472	0.074002	**0.700108**
Scared	0.670571	0.174115	0.342303	−0.08277
Joyful	−0.01537	**0.804117**	−0.13547	0.130479
Excited	0.263171	**0.754333**	0.178112	0.152586
Nervous	**0.733668**	0.187284	0.327828	−0.22283
Attentive	−0.07625	0.104636	−0.28613	**0.796554**
Active	−0.07142	0.35166	0.10899	**0.700031**
Focused	0.00085	0.029962	−0.33527	**0.809891**
Hostile	**0.800071**	0.031516	0.156503	0.002942
Aggressive	**0.885901**	0.004644	0.082433	−0.04499
Explained variance	5.479186	2.779575	2.854834	2.70483
Proportion of total	0.273959	0.138979	0.142742	0.135242

Marked loadings are >0.70.

As can be seen in Table 5, the repeated measures ANOVA shows no main effects of Group or Session nor interactions on the BAI scores, suggesting there is no effect of the AVP on anxiety scores as measured by the BAI. Similarly there were no main effects of Group or Session nor interactions on scales of Aggression and Tension. There was, however, a main Group effect on the scale of Happiness *F*_(1, 48)_ = 4.67, *p* < 0.05 with mean ratings higher for the VP Group (*M* = 6.97) as compared to the PV Group (*M* = 6.04) with no main Session effects and no interactions. There were also main Group *F*_(1, 48)_ = 4.24 *p* < 0.05 and main Session effects *F*_(1, 48)_ = 5.33 *p* < 0.05 for the scores on Focused with higher mean ratings for the VP Group (*M* = 7.61) as compared to the PV Group (*M* = 6.69) and higher scores in the first as compared to the second session (*M* = 7.45 vs. *M* = 6.85, respectively), with no Group × Session interaction. In summary, Focused and Happy ratings are higher in the VP Group as compared to the PV Group and scores of Focused are higher in the second session as compared to the first session.

**Table 5:** **Means (and *SD*) on scores on the Beck Anxiety Inventory and on selected scales of the adjusted Positive and Negative Affect Visual Analog Scale (PANAS)**.

	**Group PV (*N* = 25)**		**Group VP (*N* = 25)**	
	**Session 1 (Placebo)**	**Session 2 (Vasopressin)**	**Session 1 (Vasopressin)**	**Session 2 (Placebo)**
BAI	6.2 (5.99)	6.24 (6.31)	6.0 (6.64)	5.92 (6.24)
Aggressive	1.79 (1.29)	1.96 (1.18)	1.69 (0.83)	1.83 (1.29)
Happy^*^	6.08 (0.98)	6.04 (1.73)	6.92 (1.66)	7.02 (1.91)
Tense	2.73 (1.40)	2.98 (1.62)	3.63 (2.15)	3.16 (2.12)
Focused^**^	6.95 (1.83)	6.44 (1.57)	7.95 (1.55)	7.27 (2.23)

* *Main Group effect F_(1, 48)_ = 4.66 p = 0.036, no Session effect p > 0.8 and no interaction p > 0.6*.

** *Main Group effect F_(1, 48)_ = 4.25 p = 0.045; main session effect F_(1, 48)_ = 5.33 p < 0.05, no interaction p > 0.9*.

**Figure 2:**
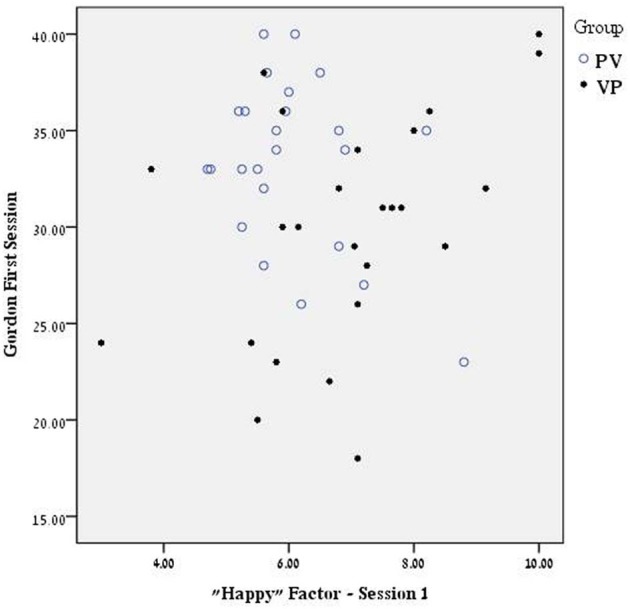
**Scatter plot showing the relationship between scores on the factor of “Happy” in the Positive and Negative Affect Scale—PANAS (Tellegen et al., 1988) and Gordon memory scores in the group receiving in the first session AVP (VP) as compared to placebo (PV).** Spearman's rank–order correlations were significant only in the VP Group (*r* = 0.43, *p* < 0.05).

In order to further examine the possible influence of these factors on the memory tests we examined the correlations (Spearman's rank-order) between the scores on these two mood scales of Happy and Focused and the four memory tests. Although there may be some carryover effects from the first to the second session, we examine here only the more easily interpretable correlations within equivalent sessions (e.g., Happy in first session and memory in first session). As seen in Table 6A, there were no such significant correlations in the group receiving Placebo in the first session (PV Group). In contrast in the VP Group that scored significantly higher on these scales, there were significant correlations between mood scores and memory scores. Remarkably these correlations reached significance only in the music tests (Table 6B). In general there is a medium-high positive correlation between both Happy and Focused scores and music memory scores (Figure 2), more noticeable in the first session (3 of 4 possible correlations are significant within this session). Those scoring high on these scales obtained higher memory scores than those who scored low on these scales.

**Table 6A:** **Spearman's rank-order correlations between verbal and music memory tests and scores on scales of Happy and Focused in Group PV (first session = placebo, second session = vasopressin)**.

	**DigitS-forward**	**DigitS-forward**	**DigitS-back**	**DigitS-back**	**MBEA**	**MBEA**	**Gordon**	**Gordon**
	**session 1** (***N* = 25**)	**session 2** (***N* = 25**)	**session 1** (***N* = 25**)	**session 2** (***N* = 25**)	**session 1** (***N* = 24**)	**session 2** (***N* = 24**)	**session 1** (***N* = 25**)	**session 2** (***N* = 25**)
Happy Session 1	0.02		−0.25		0.15		−0.08	
Happy session 2		−0.1		−0.05		−0.13		−0.23
Focused session 1	−0.27		−0.24		0.10		0.11	
Focused session 2		−0.14		0.08		0.31		0.21

**Table 6B:** **Spearman's rank-order correlations between verbal and music memory tests and scores on scales of Happy and Concentrated in Group VP (first session = vasopressin, second session = placebo)**.

	**DigitS-forward**	**DigitS-forward**	**DigitS-back**	**DigitS-back**	**MBEA**	**MBEA**	**Gordon**	**Gordon**
	**session 1** (***N* = 25**)	**session 2** (***N* = 25**)	**session 1** (***N* = 25**)	**session 2** (***N* = 25**)	**session 1** (***N* = 25**)	**session 2** (***N* = 25**)	**session 1** (***N* = 25**)	**session 2** (***N* = 25**)
Happy session 1	0.09		0.19		0.61^**^		0.43^*^	
Happy session 2		0.09		0.36		0.48^*^		0.26
Focused session 1	0.13		0.08		0.22		0.41^*^	
Focused session 2		0.17		0.14		0.43		0.30

* p < 0.05;

** p < 0.01.

In sum, the group that received vasopressin in the first session shows higher scores on scales of Happy and Focused and lower scores on the memory tests and it is only in this group that mood and memory scores are correlated, more strongly in the first session.

## Discussion

Previous genetic studies (Granot et al., 2007; Ukkola et al., 2009; Ukkola-Vuoti et al., 2011) have suggested a link between AVP and musical ability, creativity and listening habits. This link was interpreted to reflect the evolutionary roots of music as a complex vocal social communication, albeit the exact mechanisms of the presumed relationship remain to be resolved. More generally, our knowledge of the neurochemistry of musical processing is very limited with only a handful of studies which have focused on dopamine and to a lesser extent serotonin and endorphins mainly through studying brain imaging and deducing the involvement of these neurochemicals from brain imaging. In the current study we aimed at better understanding of the role of AVP in musical processing by implementing a pharmacological strategy and examining the effects of a single exposure to AVP on scores of musical memory tests. Such a methodology has been previously used to study verbal memory. This is, however, to the best of our knowledge, the first research to implement this methodology for the study of musical memory.

Our results show a complex and somewhat unexpected pattern of results. First, on the whole effects are limited to the music tests and, moreover, to the group of subjects receiving AVP in their first session. Second, contrary to the general memory enhancing effects seen in rodents, and in some human studies, in the current investigation AVP impaired memory. Third, these effects interact with the state of the subject in terms of alertness, focused attention and positive valence.

The only effect observed in the digit-span test scores was an increase in performance from the first to the second session. In contrast with the musical memory tests, no enhancing or degrading effects of AVP were observed on verbal memory. This is consistent with some previous results failing to show any effects of AVP on verbal memory (Tinklenberg and Thornton, 1983; Sahgal, 1984; Fehm-Wolfsdorf et al., 1985; Snel et al., 1987; Perras et al., 1997).

In both music tests in the first session, the group that received AVP (VP Group) performed worse than those receiving placebo (PV Group). In the second session the picture is less uniform. In the MBEA test the VP Group (which had switched to receiving Placebo in the second session) still performed worse than the PV Group, whereas in the Gordon test, there was a trend toward an interaction: The VP Group which performed significantly worse than the PV Group in the first session, improved in the second session. In contrast, the PV Group did not improve. As in the backward digit span test, this improvement may reflect some learning of strategy rather than memory for specific items, since no such learning effect was observed in the MBEA test. We do note, however, that the MBEA pounds less on WM as compared to the Gordon test. Whereas the MBEA test requires simple comparison across the two presented patterns, the Gordon test requires not only holding the presented tones in memory, comparison and shifts of attention as the sequence progresses, but also avoiding distraction and interference from the additional tones inserted as embellishing notes to create a variation on the first pattern.

A within subject design in which drug effects are tested across two sessions (one with the drug and the second with placebo) is potentially a good methodological choice since each subject serves as his or her own control eliminating between subject variability. Nonetheless, a number of studies have shown some complicated interactions related to order of treatment, which are consistent with the data reported here. For example Till and Beckwith (1985) found that desamino-D vasopressin (DDAVP) facilitated memory for sentences in the first but not in the second session. Herzmann et al. (2012) also reported a complex pattern of memory effects in the presence of oxytocin, with unexpected reduced recollection performance in the group receiving oxytocin in the first session only, with effects in the second session interpreted as related to differences in degree of proactive memory interference from session 1 to session 2. In fact, even in rodents, learning session (first, second or third), has been shown to be an important factor in AVP effects (Alescio-Lautier et al., 2000). In addition, even better researched effects such as those of cortisol on emotion, are not free from session effects as Wirth et al. (2011) conclude: “effects of cortisol on emotion vary based on situational factors, such as drug administration order or familiarity with the tasks and setting …. cortisol may only potentiate negative affect and arousal ratings in the absence of other, overwhelming influences on affect, such as the novelty of the setting and tasks in Session 1” (p. 945).

Interestingly, the degrading effects of AVP on the musical tests in the first session seem to be mediated by mood factors. Two observations were found in relation to mood. First, Happy and Focused mood scale scores were found to be higher in the VP Group as compared with the PV Group. Second, those scoring high on these scales performed better than those scoring low, but only in the presence of vasopressin (VP Group). Those receiving placebo in the first session showed no such association. A possible interpretation is that AVP interferes with musical memory but only, or more significantly in those subjects who score low on scales of positive valence (Happy), and attentiveness (Focused). This interpretation would be consistent with those models of WM stressing the role of attention as a bottleneck to WM (Cowan, 2001, 2005; Oberauer, 2002).

Selective rather than universal effects of neurohormones are not surprising given the possible interactions between individual genetic differences, difference in state and trait measures, and the complex cognitive or social tasks studied. This is clearly shown in recent studies of the effects of Oxytocin. Thus, for example, effects of oxytocin on empathic accuracy is influenced by baseline social competency (Bartz et al., 2010), with effects found only for low scorers. In another study, peripheral levels of oxytocin in mothers were found to be associated with specific traits such as sensitivity to perceptual stimulation and mood (Strathearn et al., 2012). What maybe somewhat perplexing in our results is the apparent contradiction between mean high levels of positive mood and alertness, found in the VP Group—two factors with a potential to increase cognitive performance—and the relatively low memory scores in this group. However, as Figure 2 shows, in this group but not in those receiving placebo, those scoring medium or low on positive valence and alertness are especially prone to score low on the musical memory tests, whereas those scoring especially high on these scales perform well on these tests.

Positive mood, alertness and AVP are all associated with arousal. AVP's influence on arousal, has been shown using auditory cortical ERP components related to attention, deviance detection, and orienting. This general arousal effect has been suggested by some researchers as the mediating factor in the mnemonic effects of AVP (Born et al., 1998). Interestingly, Pietrowsky et al. (1991) found that even when no such effects are seen in the electrophysiological parameters, AVP affects self-reported mood and activation similarly to what we report: Toward overall increased reported activation and alertness. Together these data suggest that AVP modulates musical memory *indirectly* through its influence on arousal and attention, though the exact modulation is far from clear. One possibility is that the degree to which this modulation is beneficial or detrimental to cognitive performance depends on the valence of the mood (Eysenck, 1982). Positive mood with arousal driven by music has been shown to enhance cognitive performance such as spatial processing (Thompson et al., 2001; Husain et al., 2002; Schellenberg et al., 2007) and may lead to better musical memory performance. Indeed attention modulation through music's ability to heighten arousal (using a pleasant upbeat children song) has been suggested as one mechanism driving beneficial effects of music on verbal memory in Alzeheimer patients (Simmons-Stern et al., 2010). In contrast negative mood with arousal may reflect stress, or result in distractibility in which case it could impair memory. In general, as presented in the Introduction most of the effects of AVP in Humans show some complex interaction between valence (e.g., response only to negative but not positive or neutral faces) and performance (e.g., identification of emotions).

One difference between the musical tests and the digit span tests that could also contribute to the different pattern of results is related to the emotional information found in music and lacking in digits. This emotional information is often associated with music's ability to serve as a social signal, moving it closer to the domain relevant to influences of AVP. Of course, in addition to this difference there are other important differences between the music and verbal tests which limit our ability to pinpoint the reason for the differences in the AVP effects on the two types of memory.

First, the digit span test is a test of recollection (subjects have to repeat verbally the sequence), whereas the music tests do not require active recollection but rather comparison (recognition). Second, digits have a visual, verbal and auditory (phonetic) representation whereas melodies, have (for non-musicians) only an auditory representation. Moreover, memory for digits could rely on explicit declarative memory, whereas memory for melodies on procedural memory. Finally, students with no musical training have much more experience with mentally manipulating digits as compared to tones.

## Conclusion

These caveats not withstanding, the current study suggests a pattern whereby AVP modulates musical WM indirectly through its ability to influence mood, attention and arousal. Interestingly music itself is a potent modulator of arousal. Ranging from lullabies and play songs used by mothers to help their babies attain an optimal level of arousal, attention and learning (Dissanayake, 2000; Malloch and Trevarthen, 2009), through meditative music, trance music, or military songs to imbue warriors with energy and courage, modulating arousal is one of music's most typical usages cross culturally (Berlyne, 1971; Clayton, 2009). Therefore, it is possible that the association found between musical aptitude (as indicated by WM scores), creativity and AVPR1a haplotypes, has to do more with individuals' susceptibility to changes in arousal and attention than with specific cognitive or social abilities. While, much more work needs to be done to verify this hypothesis, this possibility is especially interesting given the differences found here between musical and verbal WM.

### Conflict of interest statement

The authors declare that the research was conducted in the absence of any commercial or financial relationships that could be construed as a potential conflict of interest.
